# Total Energy Intake and Intake of Three Major Nutrients by Body Mass Index in Japan: NIPPON DATA80 and NIPPON DATA90

**DOI:** 10.2188/jea.JE20090219

**Published:** 2010-05-05

**Authors:** Katsushi Yoshita, Yusuke Arai, Miho Nozue, Kumi Komatsu, Hirohumi Ohnishi, Shigeyuki Saitoh, Katsuyuki Miura

**Affiliations:** 1 Project for the National Health and Nutrition Survey, Nutritional Epidemiology Program, National Institute of Health and Nutrition, Tokyo, Japan; 2 Second Department of Internal Medicine, Sapporo Medical University, Sapporo, Japan; 3 Department of Health Science, Shiga University of Medical Science, Otsu, Japan

**Keywords:** total energy intake, three major nutrients, body mass index, national nutrition survey, Japan

## Abstract

**Background:**

This paper investigated the relationship between body mass index (BMI) and total energy intake as well as intake of three major nutrients in representative Japanese populations enrolled in the National Nutrition Surveys of Japan in 1980 and 1990.

**Methods:**

A total of 10 422 participants (4585 men and 5837 women) and 8342 participants (3488 men and 4854 women) aged 30 or older from 300 randomly selected districts participated in the National Survey of Circulatory Disorders and the National Nutrition Survey in Japan in 1980 and 1990, respectively. The nutrition surveys were performed with weighing record method for three consecutive days to each household. Individually estimated total energy intake and intakes of three major nutrients (carbohydrate, protein, and fat) were compared by the categories of BMI and by 10-year age groups.

**Results:**

In men, total energy intake (kcal/day), intakes of three major nutrients (g/day) and energy intake ratio from protein and fat (%) increased as BMI increased in each age group, whereas energy intake ratio from carbohydrate (%) decreased. In women, total energy intake, intakes of three major nutrients, and energy intake ratio from protein increased as BMI increased. Energy intake ratio from carbohydrate and fat decreased as BMI increased in women in 1990. When participants were categorized into quartiles according to total energy intake in each sex group, BMI increased as total energy intake increased in men in both 1980 and 1990.

**Conclusions:**

A positive relationship was observed between body mass index and total energy intake in Japanese men. The relationship was weaker in Japanese women.

## INTRODUCTION

Proper intake of nutrients is essential for maintaining and enhancing health as well as for the prevention and treatment of diseases. Thus, in order to discuss health- and disease-related issues from a diversified perspective and to obtain reliable findings, it is important to investigate the intake of specific nutrients in target individuals and groups.^1^^,^^2^ It is of particular importance to determine the total energy intake and the intake of protein, fat and carbohydrate (three major energy nutrients), and to elucidate their possible inter-relationships as a basis for assessment of the overall nutrient intake. At the same time, it has been pointed out that assessment of body mass not only serves as an index for habitual energy intake and energy expenditure, but is also associated with the onset and progress of various lifestyle-related diseases and with mortality.^3^

Thus, in this study, we examined body mass, total energy intake and intake of three major nutrients at the time of the National Nutrition Survey, Japan (NNSJ) carried out on the subjects enrolled in the NIPPON DATA 80 and NIPPON DATA 90 studies, which are large-scale representative cohort studies of Japanese, and attempted to elucidate relationships between body mass and energy and major nutrients intake.

## METHODS

### Participants

The participants in this study were those in the National Survey on Circulatory Disorders of Japan in 1980 and 1990.^4^ Community-based participants aged 30 years and over in 300 randomly selected health districts throughout Japan participated in the survey. The numbers of participants were 10 422 (4585 men and 5837 women) in 1980 and 8342 (3488 men and 4854 women) in 1990. Cohort studies of these two populations have been named as the National Integrated Project for Prospective Observation of Non-communicable Disease and Its Trends in the Aged (NIPPON DATA80 and NIPPON DATA90). Details of NIPPON DATA80 and 90 have been described previously.^5^^–^^7^

### Nutritional survey

Detailed methods of the nutritional survey and the estimation of individual intake of nutrients and food groups were described elsewhere.^8^^–^^10^ Food intake survey by weighed food records in three consecutive representative days were conducted by specially trained dietary interviewers. Dietary interviewers visited participants’ houses at least once during the survey. Weekends and holydays were avoided. Up-dated Standard Tables for Food Composition in Japan, 4th edition, with matched fatty acid values and micronutrients, were used to calculate Japanese nutrient intakes.

Nutrient intakes of each household member were estimated by dividing household intake data of NNSJ80 and NNSJ90 conducted in 1980 and 1990, respectively, proportionally using average intakes by sex and age groups calculated for NNSJ95 conducted in 1995.^11^ The average intakes in NNSJ95 were calculated by a combination method of household-based food weighing record and an approximation of proportions by which family members shared each dish or food in the household. For each person, means of the estimated individual nutrients from the three days records were used in the analyses.

### Statistical analyses

To calculate the percentages of energy intake from protein and carbohydrate to total energy intake (protein energy ratio and carbohydrate energy ratio), intake of 1 g protein and 1 g carbohydrate was multiplied by 4 kcal. An intake of 1 g fat was multiplied by 9 kcal to calculate fat energy ratio. The total energy intake values includes energy intake from sources other than the three major nutrients, such as alcohol. Therefore, the data for many individuals does not sum to 100% when energy ratios of protein, fat and carbohydrate are added together.

Body mass index (BMI [kg/m^2^] = [weight (kg)]/[height (m)]^2^) was classified in accordance with the criteria of the Japan Society for the Study of Obesity (JASSO)^12^: “lean (or low weight)”, BMI < 18.5 (kg/m^2^); “normal (or normal weight)”, 18.5 ≤ BMI < 25.0 (kg/m^2^); and “obese (or high weight)”, BMI ≥ 25.0 (kg/m^2^).

The analysis excludes subjects whose BMI values were not obtained due to lack of data on either height, weight or both, and included individuals with a daily energy intake of between 500 kcal and 5000 kcal.

Characteristics and intakes of energy and nutrients were compared among the three categories of BMI in each of age, sex groups for NIPPON DATA80 and NIPPON DATA90, respectively. Also, mean values of BMI, height and weight were compared among the quintiles of total energy intake in men and women, separately. Analysis of variance was used to test the statistical differences among the categories.

## RESULTS

### Nutrient intakes by BMI in NIPPON DATA80

Tables 1 and 2 show nutrient intakes and characteristics by BMI groups in NIPPON DATA80 for men and women, respectively. In total men, the group with higher BMI had significantly higher values for total energy, protein, fat and carbohydrate intakes. Protein energy ratio and fat energy ratio were also higher in the higher BMI group, whereas carbohydrate energy ratio was lower. Results were almost similar in all age groups from 30–39 yeas to 70 years or over. In total women, the group with higher BMI had significantly higher values for total energy, protein, and carbohydrate intakes. Protein energy ratio was also higher in the higher BMI group, although fat energy ratio and carbohydrate energy ratio were not significantly different among BMI groups. Results were similar in all age groups of women, although total energy intake was not the highest in the obese group in women aged 60 years or over.

**Table 1.** Mean intakes of energy and nutrients according to body mass index in men: NIPPON DATA80, 1980

	Lean (BMI < 18.5 kg/m^2^)	Normal (18.5 ≤ BMI < 25.0 kg/m^2^)	Obese (BMI ≥ 25.0 kg/m^2^)	*P* value	Total
*(30–39 years)*	(*n* = 59)	(*n* = 922)	(*n* = 236)		(*n* = 1217)

Energy (kcal/day)	2282.6 ± 418.1	2467.9 ± 450.1	2530.0 ± 475.9	0.001	2471.0 ± 456.0
Protein (g/day)	81.8 ± 18.2	90.3 ± 19.3	93.1 ± 19.2	<0.001	90.5 ± 19.3
Fat (g/day)	55.6 ± 15.6	60.5 ± 18.6	63.2 ± 18.7	0.013	60.8 ± 18.6
Carbohydrate (g/day)	335.9 ± 67.2	357.3 ± 73.8	364.3 ± 77.6	0.031	357.6 ± 74.4
Protein energy ratio (%)	14.3 ± 1.7	14.7 ± 2.1	14.8 ± 1.9	0.283	14.7 ± 2.0
Fat energy ratio (%)	21.9 ± 4.3	22.0 ± 5.2	22.4 ± 4.8	0.551	22.1 ± 5.0
Carbohydrate energy ratio (%)	58.9 ± 4.7	57.9 ± 5.9	57.6 ± 5.3	0.304	57.9 ± 5.7
Age (y/o)	33.3 ± 2.6	34.2 ± 2.9	34.9 ± 2.9	<0.001	34.3 ± 2.9
Height (cm)	167.3 ± 6.0	165.9 ± 6.1	165.8 ± 5.5	0.215	166.0 ± 6.0
Body weight (kg)	49.7 ± 4.0	60.2 ± 6.4	73.7 ± 6.1	<0.001	62.3 ± 8.7
Body mass index (kg/m^2^)	17.7 ± 0.6	21.8 ± 1.7	26.8 ± 1.6	—	22.6 ± 2.8

*(40–49 years)*	(*n* = 50)	(*n* = 869)	(*n* = 276)		(*n* = 1195)

Energy (kcal/day)	2358.4 ± 415.9	2473.9 ± 452.2	2503.4 ± 447.1	0.108	2475.9 ± 450.1
Protein (g/day)	85.7 ± 15.5	92.5 ± 19.8	95.2 ± 19.9	0.005	92.9 ± 19.7
Fat (g/day)	55.1 ± 17.3	55.8 ± 17.5	58.3 ± 17.4	0.108	56.3 ± 17.5
Carbohydrate (g/day)	347.3 ± 67.1	363.0 ± 73.7	363.9 ± 73.7	0.321	362.5 ± 73.4
Protein energy ratio (%)	14.6 ± 1.6	15.0 ± 2.0	15.3 ± 2.0	0.040	15.0 ± 2.0
Fat energy ratio (%)	20.8 ± 4.8	20.2 ± 4.7	20.9 ± 4.6	0.101	20.4 ± 4.7
Carbohydrate energy ratio (%)	59.2 ± 5.8	58.7 ± 5.7	58.1 ± 6.0	0.223	58.6 ± 5.8
Age (years)	44.5 ± 3.1	44.6 ± 2.9	44.4 ± 2.9	0.414	44.6 ± 2.9
Height (cm)	164.7 ± 5.0	163.1 ± 6.1	162.8 ± 6.7	0.160	163.1 ± 6.2
Body weight (kg)	47.8 ± 3.7	59.0 ± 6.4	71.4 ± 7.0	<0.001	61.4 ± 8.8
Body mass index (kg/m^2^)	17.6 ± 1.0	22.1 ± 1.7	26.9 ± 1.8	—	23.0 ± 2.8

*(50–59 years)*	(*n* = 51)	(*n* = 763)	(*n* = 204)		(*n* = 1018)

Energy (kcal/day)	2440.1 ± 444.7	2472.3 ± 468.3	2544.6 ± 515.1	0.124	2485.1 ± 477.4
Protein (g/day)	91.2 ± 19.6	94.3 ± 20.7	98.2 ± 23.0	0.031	94.9 ± 21.1
Fat (g/day)	52.0 ± 15.7	53.0 ± 17.6	56.1 ± 18.9	0.075	53.6 ± 17.8
Carbohydrate (g/day)	372.0 ± 80.7	368.8 ± 79.2	378.3 ± 84.9	0.322	370.8 ± 80.4
Protein energy ratio (%)	15.0 ± 2.0	15.3 ± 2.2	15.5 ± 2.1	0.355	15.3 ± 2.2
Fat energy ratio (%)	19.2 ± 4.7	19.3 ± 5.1	19.8 ± 4.9	0.424	19.4 ± 5.0
Carbohydrate energy ratio (%)	60.9 ± 6.0	59.6 ± 6.4	59.4 ± 5.8	0.329	59.6 ± 6.2
Age (years)	54.6 ± 3.0	54.1 ± 2.8	53.6 ± 2.8	0.038	54.0 ± 2.8
Height (cm)	161.0 ± 6.1	160.9 ± 5.9	161.5 ± 5.4	0.409	161.1 ± 5.8
Body weight (kg)	45.7 ± 3.8	56.7 ± 6.2	70.0 ± 6.0	<0.001	58.8 ± 8.6
Body mass index (kg/m^2^)	17.6 ± 0.8	21.8 ± 1.7	26.8 ± 1.5	—	22.6 ± 2.8

*(60–69 years)*	(*n* = 67)	(*n* = 514)	(*n* = 95)		(*n* = 676)

Energy (kcal/day)	2132.7 ± 387.2	2288.1 ± 459.1	2387.9 ± 529.9	0.003	2286.7 ± 466.6
Protein (g/day)	78.5 ± 16.0	87.1 ± 18.9	91.9 ± 19.9	<0.001	86.9 ± 19.0
Fat (g/day)	42.9 ± 14.6	46.4 ± 16.4	49.5 ± 17.6	0.042	46.5 ± 16.4
Carbohydrate (g/day)	332.9 ± 62.1	354.8 ± 81.0	368.3 ± 102.0	0.027	354.5 ± 83.0
Protein energy ratio (%)	14.8 ± 1.9	15.3 ± 2.1	15.6 ± 2.3	0.065	15.3 ± 2.1
Fat energy ratio (%)	18.0 ± 4.5	18.2 ± 4.9	18.8 ± 5.2	0.535	18.3 ± 4.9
Carbohydrate energy ratio (%)	62.7 ± 6.1	62.0 ± 6.5	61.5 ± 6.5	0.501	62.0 ± 6.5
Age (years)	64.9 ± 2.8	64.2 ± 2.6	63.8 ± 2.7	0.026	64.2 ± 2.7
Height (cm)	160.0 ± 5.9	158.7 ± 6.0	160.4 ± 5.6	0.017	159.1 ± 5.9
Body weight (kg)	44.7 ± 3.6	54.6 ± 5.9	69.3 ± 6.4	<0.001	55.7 ± 8.5
Body mass index (kg/m^2^)	17.5 ± 0.9	21.6 ± 1.7	26.9 ± 1.5	—	22.0 ± 2.8

*(70– years)*	(*n* = 75)	(*n* = 341)	(*n* = 54)		(*n* = 470)

Energy (kcal/day)	1961.7 ± 387.0	1959.9 ± 405.7	2149.9 ± 407.2	0.005	1982.0 ± 406.6
Protein (g/day)	71.0 ± 15.4	73.7 ± 17.1	84.0 ± 21.2	<0.001	74.5 ± 17.7
Fat (g/day)	36.1 ± 12.8	37.8 ± 15.7	43.2 ± 16.7	0.028	38.2 ± 15.5
Carbohydrate (g/day)	322.3 ± 76.0	314.9 ± 67.8	338.6 ± 72.1	0.062	318.8 ± 69.9
Protein energy ratio (%)	14.6 ± 2.0	15.1 ± 2.2	15.6 ± 2.5	0.018	15.1 ± 2.2
Fat energy ratio (%)	16.7 ± 5.0	17.1 ± 4.8	18.1 ± 5.8	0.271	17.2 ± 4.9
Carbohydrate energy ratio (%)	65.6 ± 6.5	64.5 ± 6.7	63.0 ± 7.2	0.109	64.5 ± 6.7
Age (years)	76.2 ± 4.8	74.7 ± 4.3	74.0 ± 3.7	0.008	74.8 ± 4.4
Height (cm)	156.6 ± 4.7	156.8 ± 6.2	157.7 ± 7.4	0.533	156.9 ± 6.1
Body weight (kg)	42.7 ± 3.6	52.7 ± 6.0	66.6 ± 7.3	<0.001	52.7 ± 8.5
Body mass index (kg/m^2^)	17.4 ± 0.9	21.4 ± 1.8	26.7 ± 1.6	—	21.4 ± 2.9

*(Total)*	(*n* = 302)	(*n* = 3409)	(*n* = 865)		(*n* = 4576)

Energy (kcal/day)	2208.8 ± 441.3	2392.5 ± 478.6	2485.6 ± 487.7	<0.001	2398.0 ± 481.9
Protein (g/day)	80.6 ± 18.1	89.6 ± 20.3	94.3 ± 20.8	<0.001	89.9 ± 20.5
Fat (g/day)	47.3 ± 16.9	53.2 ± 18.8	57.2 ± 18.9	<0.001	53.6 ± 18.8
Carbohydrate (g/day)	339.8 ± 72.3	356.7 ± 76.9	366.3 ± 81.2	<0.001	357.4 ± 77.7
Protein energy ratio (%)	14.6 ± 1.8	15.1 ± 2.1	15.2 ± 2.1	<0.001	15.1 ± 2.1
Fat energy ratio (%)	19.1 ± 5.0	19.9 ± 5.2	20.7 ± 5.1	<0.001	20.0 ± 5.2
Carbohydrate energy ratio (%)	61.8 ± 6.4	59.8 ± 6.5	58.9 ± 6.1	<0.001	59.7 ± 6.4
Age (years)	56.4 ± 16.0	49.9 ± 13.4	47.9 ± 11.7	<0.001	49.9 ± 13.4
Height (cm)	161.5 ± 6.7	162.1 ± 6.8	162.8 ± 6.4	0.007	162.2 ± 6.7
Body weight (kg)	45.9 ± 4.5	57.5 ± 6.7	71.2 ± 6.7	<0.001	59.3 ± 9.2
Body mass index (kg/m^2^)	17.6 ± 0.9	21.8 ± 1.7	26.8 ± 1.6	—	22.5 ± 2.9

Values are mean ± S.D.

*P* values are by analysis of variance.

**Table 2.** Mean intakes of energy and nutrients according to body mass index in women: NIPPON DATA80, 1980

	Lean (BMI < 18.5 kg/m^2^)	Normal (18.5 ≤ BMI < 25.0 kg/m^2^)	Obese (BMI ≥ 25.0 kg/m^2^)	*P*-value	Total
*(30–39 years)*	(*n* = 121)	(*n* = 1228)	(*n* = 234)		(*n* = 1583)

Energy (kcal/day)	1886.3 ± 292.0	1960.5 ± 337.9	1975.2 ± 360.2	0.048	1957.0 ± 338.5
Protein (g/day)	70.8 ± 12.5	74.0 ± 14.9	74.4 ± 15.4	0.062	73.8 ± 14.8
Fat (g/day)	50.1 ± 13.3	52.6 ± 15.6	50.7 ± 15.7	0.068	52.1 ± 15.5
Carbohydrate (g/day)	278.6 ± 50.3	289.5 ± 56.8	296.3 ± 62.2	0.021	289.6 ± 57.3
Protein energy ratio (%)	15.0 ± 1.8	15.1 ± 1.9	15.1 ± 1.9	0.870	15.1 ± 1.9
Fat energy ratio (%)	23.9 ± 4.9	24.1 ± 5.4	23.0 ± 5.3	0.020	23.9 ± 5.4
Carbohydrate energy ratio (%)	59.1 ± 5.5	59.1 ± 5.8	60.0 ± 6.1	0.062	59.2 ± 5.8
Age (years)	33.3 ± 2.7	34.3 ± 2.9	34.9 ± 2.9	<0.001	34.3 ± 2.9
Height (cm)	154.0 ± 5.1	153.5 ± 5.1	152.0 ± 5.2	<0.001	153.3 ± 5.1
Body weight (kg)	41.8 ± 3.2	50.8 ± 5.2	63.5 ± 6.7	<0.001	52.0 ± 7.5
Body mass index (kg/m^2^)	17.6 ± 0.7	21.5 ± 1.7	27.5 ± 2.5	—	22.1 ± 3.1

*(40–49 years)*	(*n* = 72)	(*n* = 1038)	(*n* = 357)		(*n* = 1467)

Energy (kcal/day)	2050.1 ± 390.4	2015.0 ± 372.1	2046.0 ± 430.7	0.364	2024.3 ± 388.0
Protein (g/day)	76.7 ± 18.6	76.6 ± 16.3	79.7 ± 18.3	0.012	77.3 ± 16.9
Fat (g/day)	52.4 ± 15.9	51.2 ± 16.7	51.3 ± 16.8	0.834	51.3 ± 16.7
Carbohydrate (g/day)	312.0 ± 67.5	306.7 ± 63.3	312.1 ± 74.0	0.365	308.3 ± 66.3
Protein energy ratio (%)	15.0 ± 2.3	15.3 ± 2.0	15.6 ± 2.2	0.003	15.3 ± 2.1
Fat energy ratio (%)	23.0 ± 5.4	22.8 ± 5.5	22.6 ± 5.5	0.818	22.7 ± 5.5
Carbohydrate energy ratio (%)	60.9 ± 6.6	60.9 ± 6.1	61.0 ± 6.1	0.984	60.9 ± 6.1
Age (years)	44.9 ± 2.9	44.5 ± 2.9	44.9 ± 2.9	0.104	44.6 ± 2.9
Height (cm)	152.6 ± 5.3	151.8 ± 5.2	151.3 ± 5.1	0.118	151.7 ± 5.2
Body weight (kg)	40.7 ± 3.3	50.7 ± 5.2	63.8 ± 7.0	<0.001	53.4 ± 8.4
Body mass index (kg/m^2^)	17.5 ± 0.9	22.0 ± 1.7	27.8 ± 2.5	—	23.2 ± 3.4

*(50–59 years)*	(*n* = 87)	(*n* = 842)	(*n* = 387)		(*n* = 1316)

Energy (kcal/day)	1857.9 ± 385.5	1995.5 ± 397.4	1987.2 ± 394.7	0.008	1984.0 ± 397.0
Protein (g/day)	72.5 ± 15.4	77.9 ± 16.7	78.0 ± 18.0	0.016	77.6 ± 17.1
Fat (g/day)	44.2 ± 13.9	47.0 ± 15.1	46.6 ± 16.3	0.268	46.7 ± 15.4
Carbohydrate (g/day)	291.3 ± 67.9	312.8 ± 70.8	312.2 ± 70.2	0.024	311.2 ± 70.6
Protein energy ratio (%)	15.7 ± 2.0	15.7 ± 2.2	15.8 ± 2.4	0.803	15.7 ± 2.2
Fat energy ratio (%)	21.4 ± 5.0	21.2 ± 5.3	21.1 ± 5.5	0.850	21.2 ± 5.3
Carbohydrate energy ratio (%)	62.6 ± 5.9	62.7 ± 6.3	62.8 ± 6.7	0.941	62.7 ± 6.4
Age (years)	54.3 ± 2.9	54.4 ± 2.9	54.5 ± 3.0	0.684	54.4 ± 2.9
Height (cm)	150.0 ± 5.4	149.7 ± 5.1	149.1 ± 4.9	0.126	149.6 ± 5.1
Body weight (kg)	39.2 ± 3.3	49.5 ± 5.0	60.8 ± 6.0	<0.001	52.1 ± 8.1
Body mass index (kg/m^2^)	17.4 ± 0.9	22.1 ± 1.7	27.3 ± 2.0	—	23.3 ± 3.3

*(60–69 years)*	(*n* = 78)	(*n* = 602)	(*n* = 217)		(*n* = 897)

Energy (kcal/day)	1815.6 ± 383.0	1833.4 ± 379.8	1805.6 ± 413.8	0.649	1825.1 ± 388.3
Protein (g/day)	70.4 ± 19.4	71.7 ± 16.5	71.5 ± 18.9	0.835	71.5 ± 17.4
Fat (g/day)	39.7 ± 15.4	37.9 ± 14.7	39.7 ± 17.1	0.276	38.5 ± 15.3
Carbohydrate (g/day)	294.6 ± 71.2	300.7 ± 64.9	291.5 ± 68.9	0.194	298.0 ± 66.5
Protein energy ratio (%)	15.5 ± 2.1	15.7 ± 2.1	15.9 ± 2.2	0.457	15.7 ± 2.1
Fat energy ratio (%)	19.6 ± 6.1	18.4 ± 5.1	19.5 ± 5.8	0.014	18.8 ± 5.4
Carbohydrate energy ratio (%)	64.9 ± 8.1	65.8 ± 6.3	64.8 ± 7.3	0.143	65.5 ± 6.8
Age (years)	63.9 ± 2.8	64.2 ± 2.8	64.2 ± 2.8	0.695	64.2 ± 2.8
Height (cm)	149.1 ± 6.0	147.2 ± 5.4	146.5 ± 4.9	0.001	147.2 ± 5.4
Body weight (kg)	38.1 ± 4.1	47.8 ± 5.2	59.6 ± 6.2	<0.001	49.8 ± 8.2
Body mass index (kg/m^2^)	17.1 ± 1.2	22.1 ± 1.8	27.7 ± 2.2	—	23.0 ± 3.5

*(70– years)*	(*n* = 68)	(*n* = 375)	(*n* = 123)		(*n* = 566)

Energy (kcal/day)	1523.8 ± 267.6	1657.6 ± 338.5	1646.7 ± 376.1	0.011	1639.2 ± 341.7
Protein (g/day)	59.4 ± 13.1	64.3 ± 14.4	65.3 ± 19.0	0.027	63.9 ± 15.5
Fat (g/day)	30.1 ± 8.9	33.9 ± 11.9	35.7 ± 14.3	0.011	33.8 ± 12.3
Carbohydrate (g/day)	256.5 ± 53.2	276.3 ± 62.6	268.9 ± 68.4	0.047	272.3 ± 63.1
Protein energy ratio (%)	15.6 ± 2.4	15.6 ± 2.2	15.9 ± 2.4	0.573	15.7 ± 2.3
Fat energy ratio (%)	17.9 ± 4.4	18.4 ± 5.1	19.4 ± 5.8	0.077	18.5 ± 5.2
Carbohydrate energy ratio (%)	67.2 ± 5.9	66.6 ± 6.9	65.3 ± 7.7	0.115	66.4 ± 7.0
Age (years)	75.5 ± 4.7	75.5 ± 4.5	75.1 ± 4.2	0.642	75.4 ± 4.4
Height (cm)	142.4 ± 6.3	142.6 ± 6.3	142.7 ± 5.4	0.937	142.6 ± 6.1
Body weight (kg)	34.6 ± 3.5	44.5 ± 5.1	56.0 ± 6.0	<0.001	45.8 ± 8.1
Body mass index (kg/m^2^)	17.1 ± 1.2	21.9 ± 1.7	27.5 ± 2.2	—	22.5 ± 3.5

*(Total)*	(*n* = 426)	(*n* = 4085)	(*n* = 1318)		(*n* = 5829)

Energy (kcal/day)	1837.4 ± 376.4	1935.0 ± 380.6	1939.3 ± 418.2	<0.001	1928.9 ± 389.9
Protein (g/day)	70.2 ± 16.5	74.2 ± 16.3	75.6 ± 18.4	<0.001	74.2 ± 16.8
Fat (g/day)	44.2 ± 15.6	47.2 ± 16.6	46.4 ± 17.1	0.001	46.8 ± 16.7
Carbohydrate (g/day)	286.2 ± 63.7	299.1 ± 64.2	301.9 ± 70.7	<0.001	298.8 ± 65.8
Protein energy ratio (%)	15.4 ± 2.1	15.4 ± 2.0	15.6 ± 2.2	0.001	15.5 ± 2.1
Fat energy ratio (%)	21.5 ± 5.6	21.8 ± 5.7	21.4 ± 5.7	0.084	21.7 ± 5.7
Carbohydrate energy ratio (%)	62.5 ± 7.0	62.0 ± 6.7	62.4 ± 6.9	0.070	62.1 ± 6.8
Age (years)	51.9 ± 15.3	49.2 ± 13.6	51.9 ± 12.3	<0.001	50.0 ± 13.5
Height (cm)	150.2 ± 6.8	150.4 ± 6.2	149.2 ± 5.8	<0.001	150.1 ± 6.2
Body weight (kg)	39.3 ± 4.2	49.5 ± 5.5	61.5 ± 6.9	<0.001	51.5 ± 8.3
Body mass index (kg/m^2^)	17.4 ± 1.0	21.9 ± 1.7	27.6 ± 2.3	—	22.8 ± 3.4

Values are mean ± S.D.

*P* values are by analysis of variance.

### Nutrient intakes by BMI in NIPPON DATA90

Tables 3 and 4 show nutrient intakes and characteristics by BMI groups in NIPPON DATA90 for men and women, respectively. In total men of NIPPON DATA90, the group with higher BMI had significantly higher values for total energy, protein, fat and carbohydrate intakes. Protein energy ratio and fat energy ratio were also higher in the higher BMI group, whereas carbohydrate energy ratio was lower. Results were similar in each age group, although total energy intake was not the highest in the obese group of men aged 40–49 and 50–59. In total women, total energy, protein, fat and carbohydrate intakes were the lowest in the lean group; however, those in the obese group were similar to the normal weight group. Fat energy ratio was the lowest and carbohydrate energy ratio was the highest in the obese group. Total energy intake was not the highest in the obese group in women aged 40–49 and 50–59 years.

**Table 3.** Mean intakes of energy and nutrients according to body mass index in men: NIPPON DATA90, 1990

	Lean (BMI < 18.5 kg/m^2^)	Normal (18.5 ≤ BMI < 25.0 kg/m^2^)	Obese (BMI ≥ 25.0 kg/m^2^)	*P*-value	Total
*(30–39 years)*	(*n* = 31)	(*n* = 473)	(*n* = 154)		(*n* = 658)

Energy (kcal/day)	2302.0 ± 325.7	2362.9 ± 428.2	2418.9 ± 412.5	0.224	2373.1 ± 420.7
Protein (g/day)	87.2 ± 16.1	87.7 ± 17.5	90.9 ± 16.6	0.123	88.4 ± 17.3
Fat (g/day)	63.8 ± 13.6	64.9 ± 17.2	65.3 ± 16.3	0.898	64.9 ± 16.8
Carbohydrate (g/day)	314.0 ± 49.1	325.8 ± 64.8	332.8 ± 62.0	0.258	326.9 ± 63.6
Protein energy ratio (%)	15.1 ± 1.5	14.9 ± 1.7	15.1 ± 1.7	0.369	15.0 ± 1.7
Fat energy ratio (%)	24.9 ± 3.6	24.7 ± 4.2	24.3 ± 4.3	0.566	24.6 ± 4.2
Carbohydrate energy ratio (%)	54.6 ± 4.2	55.2 ± 5.1	55.1 ± 5.2	0.801	55.2 ± 5.1
Age (years)	34.4 ± 3.2	35.0 ± 2.9	35.2 ± 2.7	0.252	35.0 ± 2.9
Height (cm)	171.2 ± 6.2	168.4 ± 5.9	169.1 ± 6.3	0.026	168.7 ± 6.1
Body weight (kg)	51.8 ± 4.1	62.3 ± 6.4	77.4 ± 7.9	<0.001	65.3 ± 9.7
Body mass index (kg/m^2^)	17.7 ± 0.7	22.0 ± 1.8	27.0 ± 1.8	—	22.9 ± 3.0

*(40–49 years)*	(*n* = 24)	(*n* = 582)	(*n* = 230)		(*n* = 836)

Energy (kcal/day)	2240.3 ± 397.8	2423.2 ± 432.3	2376.5 ± 413.6	0.059	2405.1 ± 427.2
Protein (g/day)	85.6 ± 21.3	93.5 ± 18.2	92.8 ± 17.2	0.108	93.1 ± 18.0
Fat (g/day)	59.7 ± 17.3	62.8 ± 15.6	60.0 ± 15.0	0.062	61.9 ± 15.5
Carbohydrate (g/day)	303.0 ± 49.6	331.2 ± 67.0	329.2 ± 63.7	0.117	329.9 ± 65.8
Protein energy ratio (%)	15.3 ± 2.6	15.5 ± 1.8	15.7 ± 1.9	0.298	15.6 ± 1.9
Fat energy ratio (%)	23.7 ± 3.6	23.3 ± 4.0	22.7 ± 3.6	0.090	23.2 ± 3.9
Carbohydrate energy ratio (%)	54.5 ± 5.6	54.7 ± 5.0	55.4 ± 4.6	0.141	54.9 ± 4.9
Age (years)	44.0 ± 2.9	44.0 ± 2.9	44.4 ± 3.1	0.159	44.1 ± 3.0
Height (cm)	167.9 ± 6.8	166.3 ± 5.9	165.9 ± 5.3	0.205	166.2 ± 5.8
Body weight (kg)	50.0 ± 4.0	61.6 ± 6.4	74.4 ± 6.8	<0.001	64.8 ± 9.0
Body mass index (kg/m^2^)	17.7 ± 0.6	22.2 ± 1.7	27.0 ± 1.9	—	23.4 ± 2.9

*(50–59 years)*	(*n* = 30)	(*n* = 551)	(*n* = 211)		(*n* = 792)

Energy (kcal/day)	2327.5 ± 341.7	2458.2 ± 493.1	2434.3 ± 441.7	0.308	2446.9 ± 475.2
Protein (g/day)	87.1 ± 11.9	97.3 ± 20.1	97.6 ± 22.6	0.028	97.0 ± 20.6
Fat (g/day)	54.1 ± 11.4	59.9 ± 17.1	60.0 ± 17.3	0.187	59.7 ± 17.0
Carbohydrate (g/day)	340.0 ± 54.1	344.7 ± 76.9	340.9 ± 66.2	0.783	343.5 ± 73.4
Protein energy ratio (%)	15.1 ± 2.0	15.9 ± 1.9	16.0 ± 2.1	0.060	15.9 ± 2.0
Fat energy ratio (%)	20.9 ± 3.0	21.9 ± 4.2	22.1 ± 4.3	0.312	21.9 ± 4.2
Carbohydrate energy ratio (%)	58.5 ± 4.3	56.1 ± 5.4	56.2 ± 5.8	0.070	56.2 ± 5.5
Age (years)	56.0 ± 2.2	54.5 ± 2.9	54.7 ± 2.9	0.024	54.7 ± 2.9
Height (cm)	161.8 ± 5.6	162.7 ± 5.9	163.0 ± 5.6	0.559	162.8 ± 5.8
Body weight (kg)	46.5 ± 3.4	59.0 ± 6.2	71.1 ± 6.8	<0.001	61.8 ± 8.8
Body mass index (kg/m^2^)	17.8 ± 0.6	22.3 ± 1.6	26.7 ± 1.8	—	23.3 ± 2.8

*(60–69 years)*	(*n* = 58)	(*n* = 512)	(*n* = 138)		(*n* = 708)

Energy (kcal/day)	2196.9 ± 512.2	2222.9 ± 395.6	2310.3 ± 429.7	0.065	2237.8 ± 414.0
Protein (g/day)	84.1 ± 19.0	86.8 ± 17.7	89.8 ± 19.1	0.096	87.2 ± 18.1
Fat (g/day)	52.6 ± 19.8	52.3 ± 15.7	54.7 ± 14.9	0.291	52.8 ± 15.9
Carbohydrate (g/day)	316.3 ± 84.0	323.4 ± 63.4	336.9 ± 72.2	0.061	325.4 ± 67.2
Protein energy ratio (%)	15.4 ± 2.0	15.7 ± 1.9	15.6 ± 1.9	0.644	15.6 ± 1.9
Fat energy ratio (%)	21.3 ± 5.3	21.0 ± 4.5	21.3 ± 4.6	0.765	21.1 ± 4.6
Carbohydrate energy ratio (%)	57.7 ± 6.5	58.3 ± 5.8	58.3 ± 5.5	0.754	58.3 ± 5.8
Age (years)	64.3 ± 2.8	64.1 ± 2.8	64.1 ± 2.7	0.856	64.1 ± 2.8
Height (cm)	162.3 ± 6.8	160.9 ± 5.8	160.6 ± 5.9	0.148	160.9 ± 5.9
Body weight (kg)	45.8 ± 4.1	57.1 ± 6.3	70.0 ± 8.2	<0.001	58.7 ± 9.1
Body mass index (kg/m^2^)	17.4 ± 0.9	22.0 ± 1.7	27.1 ± 2.1	—	22.6 ± 3.1

*(70– years)*	(*n* = 65)	(*n* = 342)	(*n* = 82)		(*n* = 489)

Energy (kcal/day)	1974.9 ± 399.7	1979.3 ± 407.6	2016.7 ± 455.3	0.747	1985.0 ± 414.3
Protein (g/day)	75.4 ± 16.7	77.7 ± 17.6	80.0 ± 19.4	0.303	77.8 ± 17.8
Fat (g/day)	44.4 ± 14.6	44.7 ± 14.4	46.9 ± 15.4	0.445	45.0 ± 14.6
Carbohydrate (g/day)	302.8 ± 65.4	299.4 ± 67.2	299.5 ± 67.7	0.929	299.9 ± 66.9
Protein energy ratio (%)	15.3 ± 1.9	15.8 ± 2.2	15.9 ± 2.0	0.213	15.7 ± 2.1
Fat energy ratio (%)	20.2 ± 4.9	20.3 ± 4.5	20.7 ± 4.3	0.678	20.3 ± 4.5
Carbohydrate energy ratio (%)	61.4 ± 5.8	60.5 ± 6.2	59.8 ± 6.5	0.269	60.5 ± 6.2
Age (years)	76.6 ± 4.9	75.8 ± 4.6	74.4 ± 4.6	0.011	75.7 ± 4.7
Height (cm)	159.3 ± 6.6	157.8 ± 6.1	159.5 ± 5.7	0.025	158.3 ± 6.2
Body weight (kg)	43.9 ± 4.3	54.2 ± 6.1	68.4 ± 6.2	<0.001	55.2 ± 9.0
Body mass index (kg/m^2^)	17.3 ± 0.9	21.7 ± 1.8	26.8 ± 1.4	—	22.0 ± 3.1

*(Total)*	(*n* = 208)	(*n* = 2460)	(*n* = 815)		(*n* = 3483)

Energy (kcal/day)	2167.0 ± 435.7	2316.0 ± 463.2	2352.1 ± 443.3	<0.001	2315.6 ± 458.6
Protein (g/day)	82.5 ± 17.8	89.6 ± 19.3	91.9 ± 19.7	<0.001	89.7 ± 19.4
Fat (g/day)	52.8 ± 17.3	57.8 ± 17.5	58.8 ± 16.6	<0.001	57.7 ± 17.3
Carbohydrate (g/day)	313.6 ± 66.7	327.2 ± 69.5	331.2 ± 66.8	0.004	327.3 ± 68.8
Protein energy ratio (%)	15.3 ± 2.0	15.6 ± 1.9	15.7 ± 2.0	0.032	15.6 ± 1.9
Fat energy ratio (%)	21.7 ± 4.7	22.4 ± 4.5	22.4 ± 4.3	0.110	22.3 ± 4.5
Carbohydrate energy ratio (%)	58.2 ± 6.1	56.7 ± 5.8	56.5 ± 5.6	0.001	56.7 ± 5.8
Age (years)	60.1 ± 15.3	53.2 ± 13.8	51.7 ± 12.5	<0.001	53.3 ± 13.7
Height (cm)	163.3 ± 7.7	163.6 ± 6.9	164.2 ± 6.5	0.064	163.7 ± 6.9
Body weight (kg)	46.7 ± 4.9	59.2 ± 6.9	72.8 ± 7.8	<0.001	61.6 ± 9.8
Body mass index (kg/m^2^)	17.5 ± 0.8	22.1 ± 1.7	26.9 ± 1.9	—	22.9 ± 3.0

Values are mean ± S.D.

*P* values are by analysis of variance.

**Table 4.** Mean intakes of energy and nutrients according to body mass index in women: NIPPON DATA90, 1990

	Lean (BMI < 18.5 kg/m^2^)	Normal (18.5 ≤ BMI < 25.0 kg/m^2^)	Obese (BMI ≥ 25.0 kg/m^2^)	*P* value	Total
*(30–39 years)*	(*n* = 103)	(*n* = 788)	(*n* = 140)		(*n* = 1031)

Energy (kcal/day)	1850.1 ± 300.7	1872.7 ± 315.5	1944.5 ± 308.3	0.026	1880.2 ± 313.9
Protein (g/day)	72.5 ± 13.4	71.3 ± 13.0	73.7 ± 13.1	0.109	71.7 ± 13.1
Fat (g/day)	57.0 ± 13.1	57.1 ± 14.0	58.0 ± 14.7	0.778	57.2 ± 14.0
Carbohydrate (g/day)	254.3 ± 44.7	260.9 ± 48.8	274.5 ± 47.5	0.002	262.1 ± 48.5
Protein energy ratio (%)	15.7 ± 1.8	15.3 ± 1.7	15.2 ± 1.8	0.038	15.3 ± 1.7
Fat energy ratio (%)	27.7 ± 4.5	27.4 ± 4.3	26.7 ± 4.6	0.173	27.3 ± 4.4
Carbohydrate energy ratio (%)	55.1 ± 4.9	55.8 ± 4.8	56.6 ± 5.3	0.058	55.8 ± 4.9
Age (years)	34.1 ± 2.6	34.9 ± 2.8	34.9 ± 2.8	0.027	34.8 ± 2.8
Height (cm)	156.3 ± 5.6	155.9 ± 5.1	154.2 ± 5.3	0.001	155.7 ± 5.2
Body weight (kg)	43.1 ± 3.5	51.9 ± 5.0	65.1 ± 6.7	<0.001	52.8 ± 7.5
Body mass index (kg/m^2^)	17.6 ± 0.7	21.4 ± 1.7	27.3 ± 2.2	—	21.8 ± 3.0

*(40–49 years)*	(*n* = 59)	(*n* = 867)	(*n* = 244)		(*n* = 1170)

Energy (kcal/day)	1929.3 ± 303.1	1970.8 ± 348.9	1953.8 ± 366.2	0.578	1965.1 ± 350.3
Protein (g/day)	76.1 ± 14.2	78.4 ± 15.3	78.2 ± 15.6	0.550	78.2 ± 15.3
Fat (g/day)	55.8 ± 12.2	56.7 ± 14.4	55.2 ± 14.4	0.349	56.4 ± 14.3
Carbohydrate (g/day)	273.3 ± 49.7	280.1 ± 55.2	279.6 ± 57.8	0.660	279.7 ± 55.5
Protein energy ratio (%)	15.8 ± 2.0	16.0 ± 1.9	16.1 ± 2.0	0.549	16.0 ± 1.9
Fat energy ratio (%)	26.1 ± 4.8	25.8 ± 4.2	25.4 ± 4.1	0.282	25.8 ± 4.2
Carbohydrate energy ratio (%)	56.6 ± 5.2	56.9 ± 5.1	57.2 ± 5.0	0.569	56.9 ± 5.1
Age (years)	43.8 ± 3.2	44.1 ± 2.9	45.1 ± 2.9	<0.001	44.3 ± 3.0
Height (cm)	154.8 ± 5.3	153.6 ± 5.2	152.6 ± 4.8	0.005	153.4 ± 5.2
Body weight (kg)	42.3 ± 3.5	51.4 ± 5.2	64.4 ± 6.9	<0.001	53.7 ± 8.1
Body mass index (kg/m^2^)	17.6 ± 0.8	21.8 ± 1.7	27.6 ± 2.4	—	22.8 ± 3.2

*(50–59 years)*	(*n* = 40)	(*n* = 694)	(*n* = 298)		(*n* = 1032)

Energy (kcal/day)	1918.4 ± 387.7	1928.5 ± 351.5	1925.6 ± 401.8	0.982	1927.3 ± 367.7
Protein (g/day)	76.5 ± 14.2	78.1 ± 15.6	78.7 ± 18.8	0.714	78.2 ± 16.5
Fat (g/day)	51.2 ± 13.4	51.7 ± 14.3	50.4 ± 15.6	0.415	51.3 ± 14.6
Carbohydrate (g/day)	281.7 ± 66.0	285.4 ± 59.3	287.9 ± 63.8	0.760	286.0 ± 60.8
Protein energy ratio (%)	16.1 ± 2.1	16.3 ± 2.0	16.4 ± 2.0	0.655	16.3 ± 2.0
Fat energy ratio (%)	24.0 ± 4.5	24.1 ± 4.7	23.4 ± 4.8	0.127	23.9 ± 4.7
Carbohydrate energy ratio (%)	58.7 ± 5.4	59.2 ± 5.7	60.0 ± 6.2	0.114	59.4 ± 5.8
Age (years)	54.6 ± 2.9	54.5 ± 2.8	54.7 ± 2.8	0.543	54.5 ± 2.8
Height (cm)	151.5 ± 7.2	151.4 ± 5.1	151.3 ± 5.2	0.992	151.4 ± 5.2
Body weight (kg)	39.7 ± 4.4	50.6 ± 5.0	62.3 ± 6.5	<0.001	53.6 ± 8.1
Body mass index (kg/m^2^)	17.3 ± 1.0	22.1 ± 1.7	27.2 ± 2.2	—	23.4 ± 3.2

*(60–69 years)*	(*n* = 64)	(*n* = 564)	(*n* = 287)		(*n* = 915)

Energy (kcal/day)	1749.1 ± 323.4	1810.5 ± 367.6	1818.9 ± 398.5	0.397	1808.9 ± 374.7
Protein (g/day)	68.7 ± 14.3	72.6 ± 15.2	73.5 ± 18.5	0.110	72.6 ± 16.2
Fat (g/day)	43.8 ± 13.1	45.8 ± 14.0	44.1 ± 14.5	0.210	45.1 ± 14.1
Carbohydrate (g/day)	267.8 ± 53.9	276.2 ± 63.4	281.0 ± 67.8	0.281	277.1 ± 64.3
Protein energy ratio (%)	15.8 ± 1.8	16.1 ± 2.1	16.2 ± 2.3	0.315	16.1 ± 2.1
Fat energy ratio (%)	22.5 ± 4.9	22.7 ± 4.8	21.7 ± 5.0	0.023	22.4 ± 4.8
Carbohydrate energy ratio (%)	61.3 ± 5.6	61.1 ± 6.3	61.9 ± 6.5	0.191	61.3 ± 6.3
Age (years)	65.5 ± 2.6	63.9 ± 2.8	64.2 ± 2.9	<0.001	64.1 ± 2.8
Height (cm)	148.8 ± 5.2	148.7 ± 5.8	148.3 ± 5.4	0.523	148.6 ± 5.6
Body weight (kg)	38.2 ± 3.1	49.0 ± 5.4	60.5 ± 6.7	<0.001	51.8 ± 8.6
Body mass index (kg/m^2^)	17.3 ± 0.8	22.1 ± 1.7	27.5 ± 2.4	—	23.5 ± 3.6

*(70– years)*	(*n* = 71)	(*n* = 446)	(*n* = 182)		(*n* = 699)

Energy (kcal/day)	1576.4 ± 306.5	1609.0 ± 327.6	1646.9 ± 329.0	0.236	1615.5 ± 326.1
Protein (g/day)	61.8 ± 14.5	64.1 ± 13.9	66.3 ± 14.9	0.053	64.4 ± 14.3
Fat (g/day)	37.0 ± 10.3	38.7 ± 12.5	37.6 ± 11.5	0.377	38.3 ± 12.0
Carbohydrate (g/day)	249.5 ± 54.7	250.9 ± 54.8	260.2 ± 58.8	0.143	253.2 ± 55.9
Protein energy ratio (%)	15.7 ± 1.9	16.0 ± 2.0	16.2 ± 2.2	0.242	16.0 ± 2.1
Fat energy ratio (%)	21.3 ± 4.9	21.5 ± 4.5	20.6 ± 4.9	0.066	21.2 ± 4.7
Carbohydrate energy ratio (%)	63.2 ± 5.3	62.5 ± 6.1	63.2 ± 6.3	0.373	62.7 ± 6.0
Age (years)	76.6 ± 5.2	76.2 ± 4.9	75.3 ± 4.8	0.073	76.0 ± 4.9
Height (cm)	144.3 ± 5.7	144.3 ± 6.2	144.2 ± 6.7	0.968	144.3 ± 6.3
Body weight (kg)	35.7 ± 3.4	45.6 ± 5.3	56.6 ± 6.5	<0.001	47.5 ± 8.3
Body mass index (kg/m^2^)	17.1 ± 1.1	21.8 ± 1.8	27.2 ± 1.8	—	22.8 ± 3.4

*(Total)*	(*n* = 337)	(*n* = 3359)	(*n* = 1151)		(*n* = 4847)

Energy (kcal/day)	1795.2 ± 341.1	1864.1 ± 360.6	1863.2 ± 386.6	0.004	1859.1 ± 366.0
Protein (g/day)	70.6 ± 14.9	73.8 ± 15.4	74.7 ± 17.4	<0.001	73.8 ± 15.9
Fat (g/day)	49.4 ± 14.7	51.5 ± 15.4	48.8 ± 15.9	<0.001	50.7 ± 15.5
Carbohydrate (g/day)	262.4 ± 53.2	272.2 ± 57.3	278.4 ± 61.6	<0.001	273.0 ± 58.2
Protein energy ratio (%)	15.8 ± 1.9	15.9 ± 2.0	16.1 ± 2.1	0.006	15.9 ± 2.0
Fat energy ratio (%)	24.7 ± 5.3	24.7 ± 4.9	23.4 ± 5.1	<0.001	24.4 ± 5.0
Carbohydrate energy ratio (%)	58.7 ± 6.1	58.5 ± 6.0	60.0 ± 6.4	<0.001	58.9 ± 6.1
Age (years)	53.2 ± 16.9	51.7 ± 14.0	55.9 ± 12.9	<0.001	52.8 ± 14.1
Height (cm)	151.5 ± 7.3	151.6 ± 6.5	150.1 ± 6.3	<0.001	151.2 ± 6.6
Body weight (kg)	40.1 ± 4.5	50.2 ± 5.5	61.8 ± 7.2	<0.001	52.2 ± 8.3
Body mass index (kg/m^2^)	17.4 ± 0.9	21.8 ± 1.7	27.4 ± 2.2	—	22.8 ± 3.3

Values are mean ± S.D.

*P* values are by analysis of variance.

### Nutrient-specific energy intake by sex and BMI

**Figure 1.**
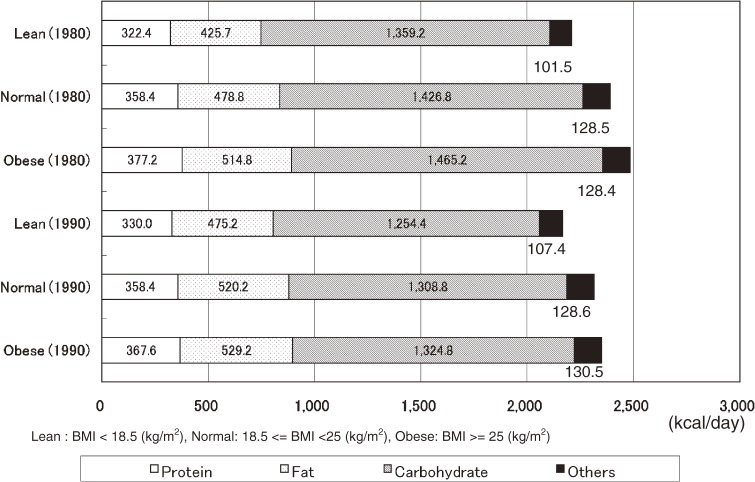
Mean energy intakes from protein, fat, and carbohydrate according to body mass index in men: NIPPON DATA80 (1980) and NIPPON DATA90 (1990)

**Figure 2.**
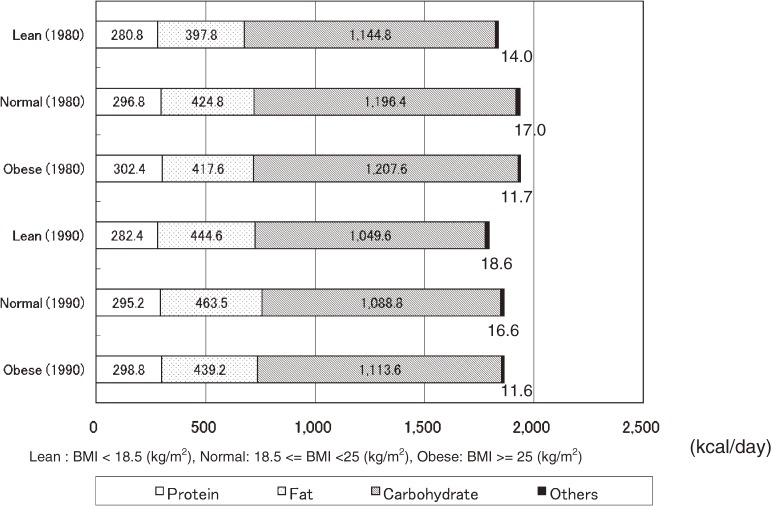
Mean energy intakes from protein, fat, and carbohydrate according to body mass index in women: NIPPON DATA80 (1980) and NIPPON DATA90 (1990)

Figure 1 and Figure 2 show nutrient-specific energy intake by sex and BMI in NIPPON DATA80 and NIPPON DATA90. In men, 100–130 kcal/day of energy intake came from sources other than the three major nutrients. In women, 11–19 kcal/day of energy intake came from sources other than the three major nutrients.

## DISSCUSSION

The results obtained in this study show that in men in both survey years, total energy increases from low body weight/low BMI to high weight/high BMI. On the other hand, when looking into the results for 1990 in women and the details by sex/age, such a tendency was not necessarily observed in all cases. This may possibly be attributed to the fact that the number of evaluable subjects decreased and a characteristic of Japanese women whereby they intentionally reduce the amount of food intake during a survey period due to wanting to be defined as a small eater. Nonetheless, we have concluded that there is an overall tendency towards those with high BMI having high total energy intake.

We then categorized total energy intake by sex in each year into the quartiles to see the relationship with BMI, and found that, in men, the group with higher total energy intake had higher BMI in both 1980 and 1980. This indicates the existence of the same relationship in the opposite direction as the aforementioned relationship with the total energy intake viewed from BMI. However, the same result was not obtained in women. Although the reason is not entirely clear, it is considered that the drive for slimness^17^ commonly found in adolescent women in Japan that is associated with the aforementioned reason may possibly have an impact on this result.

In the last twenty years, the prevalence of obesity among Japanese male adults has increased in every age group and there is an associated risk of increases in the number of patients with diabetes, hypertension, hyperlipidemia, or more serious diseases such as ischemic heart disease and stroke. On the other hand, in Japanese female adults, the prevalence of obesity decreases with younger age, with an increasing number of female young adults classified as “lean”.^13^ Low weight in early life does not simply mean that energy intake is lower than appropriate, but it poses an increased risk of diseases caused by low intake of the three major nutrients, vitamins and minerals, due to low overall food intake. Also, we face such issues as an increase in the number of low-birth weight infants due to insufficient nutrient intake during pregnancy, and an increase in the associated risks, such as metabolic syndrome in their future life.^14^^,^^15^ Thus, an examination of body mass, energy and nutrient intake and the balance of intake of the three major nutrient types may provide important clues to the prevention and treatment of various diseases, primarily lifestyle-related diseases. Changes in weight in healthy adults can serve as an objective indicator of the relationship between usual energy intake and energy expenditure during a given time period.^16^ In other words, if the energy balance is positive, body weight will increase and if the balance is negative, it will decline. It is our interpretation that weight and body mass are defined at a given time during the long term in which changes in weight take place. Therefore, some relevance is attached to the energy intake defined by this study if a higher energy intake is observed in people with higher BMI when moving from the lean group to the obese group, assuming that the average level of physical activity and related conditions do not differ significantly between groups.

The nutrient intake surveys used in this study were obtained from the National Nutrition Survey, Japan conducted nationwide in 1980 and 1990. In between these two survey years, the food database was drastically renewed, as was the survey method.^8^^,^^9^ From this, one might argue that it is problematic to simply compare the results of the two years. Nevertheless, it is generally observed that the total energy intake in 1990 is on a declining trend compared with 1980. However, fat energy ratio tended to be higher in total participants for both men and women in 1990 compared with that in 1980. Thus, the fat energy ratio increased by about 1–3% during the 10 years. This tendency seems to indicate the process of transition of the average dietary patterns of the Japanese from the traditional Japanese diet to a more western diet.^13^

Energy intake from nutrients other than the three major nutrients accounted for 100–130 kcal in men, much of which is assumed to be energy intake derived from alcohol. Japanese men are ranked as consuming a relatively high volume of alcohol compared with other major countries and an association with various diseases, including hypertension, has been reported.^18^ Since excessive alcohol consumption has a large impact on the customary nutrient intakes and their ratios,^19^^,^^20^ detailed analyses from this perspective are warranted in the future. A report by Ueda, et al studied the relationship between obesity and the nutrient intake survey results in men aged 40–59 years obtained by the INTERMAP Study conducted in Japan, and found that there was a significant positive relationship between fat energy ratio and BMI, being independent from other factors.^21^ The results of this study demonstrate a similar tendency.

Because the results in this study have been estimated based on the data of the National Nutrition Survey, Japan in 1980 and 1990, it may not be entirely appropriate to treat the obtained knowledge as a precise indicator of the current state of nutrition in Japan. Nonetheless, the results in this study are highly likely to be useful for elucidating in a multilateral manner the relationships between various risk factors, including those of cardiovascular diseases, related data or death and nutrition/diet. These efforts will be further enhanced by linking these findings with follow-up data from future NIPPON DATA studies, and hence we expect further exploration of the relationship between nutrient intake and body mass in the Japanese population.

## References

[1] Barasi ME, Nutritional principles. In: Nutrition at a Glance. Oxford, UK; 2007. p. 6–25.

[2] Gidding SS , Lichtenstein AH , Faith MS , Karpyn A , Mennella JA , Popkin B , Implementing American Heart Association Pediatric and Adult Nutrition Guidelines: a scientific statement from the American Heart Association Nutrition Committee of the Council on Nutrition, Physical Activity and Metabolism, Council on Cardiovascular Disease in the Young, Council on Arteriosclerosis, Thrombosis and Vascular Biology, Council on Cardiovascular Nursing, Council on Epidemiology and Prevention, and Council for High Blood Pressure Research . Circulation. 2009;119:1161–75 10.1161/CIRCULATIONAHA.109.191856 19255356

[3] Funada S , Shimazu T , Kakizaki M , Kuriyama S , Sato Y , Matsuda-Ohmori K , Body mass index and cardiovascular disease mortality in Japan: the Ohsaki Study . Prev Med. 2008;47:66–70 10.1016/j.ypmed.2008.03.010 18462784

[4] Japanese Ministry of Health and Welfare. National Survey on Circulatory Disorders (in Japanese). Japan Heart Foundation, Tokyo; 1982.

[5] Ueshima H NIPPON DATA . Nippon Rinsho. 2006;64 Suppl 6:108–11 (in Japanese) 16981529

[6] Ueshima H , Choudhury SR , Okayama A , Hayakawa T , Kita Y , Kadowaki T , Cigarette smoking as a risk factor for stroke death in Japan: NIPPON DATA80 . Stroke. 2004;35:1836–41 10.1161/01.STR.0000131747.84423.74 15166389

[7] Kadota A , Hozawa A , Okamura T , Kadowak T , Nakmaura K , Murakami Y , ; NIPPON DATA Research Group Relationship between metabolic risk factor clustering and cardiovascular mortality stratified by high blood glucose and obesity: NIPPON DATA90, 1990–2000 . Diabetes Care. 2007;30:1533–8 10.2337/dc06-2074 17363755

[8] Ministry of Health and Welfare. The National Nutrition Survey in Japan, 1980. Tokyo: Daiichi Shuppan; 1982 (in Japanese).

[9] Ministry of Health and Welfare. The National Nutrition Survey in Japan, 1990. Tokyo: Daiichi Shuppan; 1992 (in Japanese).

[10] Okuda N , Miura K , Yoshita K , Matsumura Y , Nakamura Y , Okayama A , Integration of data from NIPPON DATA80/90 and National Nutrition Survey in Japan: for cohort studies of representative Japanese on nutrition . J Epidemiol. 2010;20 Suppl 3;S506–14 10.2188/jea.JE20090218 20351471 PMC3920387

[11] Ministry of Health and Welfare. The National Nutrition Survey in Japan, 1995. Tokyo: Daiichi Shuppan; 1997 (in Japanese).

[12] Japan Society for the Study of Obesity Guidelines for the management of obesity 2006 . Journal of Japan Society for the Study of Obesity. 2006;12:10–5 (in Japanese)

[13] Ministry of Health. Labour and Welfare. The National Health and Nutrition Survey in Japan, 2007. Tokyo: Daiichi Shuppan; 2009 (in Japanese).

[14] Barker DJ , Hales CN , Fall CH , Osmond C , Phipps K , Clark PM Type 2 (non-insulin-dependent) diabetes mellitus, hypertension and hyperlipidaemia (syndrome X): relation to reduced fetal growth . Diabetologia. 1993;36:62–7 10.1007/BF00399095 8436255

[15] Barker DJ , Osmond C , Simmonds SJ , Wield GA The relation of small head circumference and thinness at birth to death from cardiovascular disease in adult life . BMJ. 1993;306:422–6 10.1136/bmj.306.6875.422 8461722 PMC1676496

[16] Ministry of Health, Labour and Welfare. Dietary Reference Intakes for Japanese (2010 ed.). Daiichi Shuppan; 2009 (in Japanese).

[17] Hayashi F , Takimoto H , Yoshita K , Yoshiike N Perceived body size and desire for thinness of young Japanese women: a population-based survey . Br J Nutr. 2006;96:1154–62 10.1017/BJN20061921 17181892

[18] Yoshita K , Miura K , Morikawa Y , Ishizaki M , Kido T , Naruse Y , Relationshiphip of alcohol consumption to 7-year blood pressure change in Japanese men . J Hypertens. 2005;23:1485–90 10.1097/01.hjh.0000173394.39197.4e 16003174

[19] Yoshita K The relationshiphips between alcohol, Food and nutrient intakes and health examination results . Journal of the Japanese Association for Cerebro-cardiovascular Disease Control. 1998;33:186–98 (in Japanese)

[20] Yoshita K , Tabata M , Takase E , Kadoshima Y , Ishizaki M , Miura K , The relationshiphips between alcohol intake and contribution ratio of energy intake . Hokuriku Journal of Public Health. 1999;26:34–7 (in Japanese)

[21] Ueda H , Higashiyama A , Okayama A , Okamura T , Okuda N , Yoshita K , Obesity and percentage energy from fat: The intermap study of middle-aged Japanese men . Japanese Journal of Cardiovascular Disease Prevention. 2008;43:123–31 (in Japanese)

